# Impact of an augmented reality-based decision support system on teamwork, leadership, provider workload and cognitive load during simulated cardiac arrest – a simulation-based randomized controlled trial

**DOI:** 10.1186/s41077-026-00444-9

**Published:** 2026-05-01

**Authors:** Adam Cheng, Sergio Manzano, Johan N. Siebert, Alexandre De Masi, Jennifer Davidson, Kangsoo Kim, Frédéric Ehrler, Delphine S. Courvoisier, Donovan Duncan, Ana Rajic, Sharleen K. Olanka, Ryan Kang, Yiqun Lin, the InterFACE-AR Clinical Investigators

**Affiliations:** 1 Departments of Pediatrics and Emergency Medicine, Cumming School of Medicine, KidSIM Simulation Program, Alberta Children’s Hospital, University of Calgary, 28 Oki Drive NW, Calgary, AB T3B 6A8 Canada; 2 Department of Pediatric Emergency Medicine, Geneva Children’s Hospital, Geneva University Hospitals, Faculty of Medicine, University of Geneva, Geneva, Switzerland; 3 Faculty of Medicine, University of Geneva, Geneva, Switzerland; 4 Cumming School of Medicine, KidSIM Simulation Program, Alberta Children’s Hospital, University of Calgary, Calgary, Canada; 5 Department of Electrical and Software Engineering, Schulich School of Engineering, University of Calgary, Calgary, Canada; 6 Division of Computer Sciences, Geneva University Hospitals, Faculty of Medicine, University of Geneva, Geneva, Switzerland; 7 Quality of Care Division, Geneva University Hospitals, Faculty of Medicine, University of Geneva, Geneva, Switzerland; 8 Department of Pediatrics, Cumming School of Medicine, University of Calgary, Calgary, Canada; 9 Educational Technologies and Learning Sciences (TECFA), Faculty of Psychology and Educational Sciences, University of Geneva, Geneva, Switzerland; 10 KidSIM Simulation Program, Alberta Children’s Hospital, Calgary, Canada

**Keywords:** Cardiopulmonary resuscitation, Cognitive aid, Augmented reality, Teamwork, Cognitive load, Workload, Leadership

## Abstract

**Background:**

In cardiac arrest management, cognitive aids provide prompts to encourage recall of critical information, which may improve clinical performance. Whether cognitive aids influence provider workload, cognitive load, teamwork dynamics, or leadership during cardiac arrest remains unknown. In this study, we evaluated the effect of using a multi-faceted decision support system with augmented reality-based cognitive aids (i.e. InterFACE-AR) vs. the American Heart Association (AHA) Pediatric Advanced Life Support (PALS) pocket card on provider workload and cognitive load, teamwork, and leadership during simulated pediatric cardiac arrest.

**Methods:**

We conducted secondary analysis of data collected from a prospective, randomized controlled trial comparing the use of the InterFACE-AR system to the AHA PALS pocket card during simulated pediatric cardiac arrest. Participants were recruited in groups of 3 to perform the roles of team leader, medication nurse, and documenting nurse. All teams completed a 12-min simulated cardiac arrest scenario. Provider workload (NASA-RTLX) and cognitive load (Paas score) were captured from participants after the scenario. Teamwork (TEAM score) and leadership performance (CALM score) were assessed via video review.

**Results:**

A total of 18 simulation sessions were analyzed (Control: *n* = 9; InterFACE-AR: *n* = 9), involving 54 participants in total. Team leaders using the InterFACE-AR system had lower RTLX (mean difference [MD]: -15.0; 95% confidence interval [CI]: -27.0 to -4.6, *p* = 0.022) and Paas score (MD: -2.4; 95%CI: -3.6 to -1.4, *p* < 0.001), while documenting nurses showed similar reductions (RTLX -13.7, 95%CI: -26.7 to -0.4, *p* = 0.049; Paas -1.6, 95%CI: -2.8 to -0.1, *p* = 0.046) compared with those using PALS pocket card. Medication nurses demonstrated no statistically significant differences in RTLX (*p* = 0.098) or Paas score (*p* = 0.194). Teams using the InterFACE-AR system achieved significantly higher TEAM scores compared to those using PALS pocket card only (39.2 vs 35.8, MD: 3.4, 95%CI: 0.8 – 5.9, *p* = 0.030). CALM scores did not differ significantly between groups.

**Conclusion:**

Use of an AR-based decision support system during simulated pediatric cardiac arrest reduces workload and cognitive load for the team leader and documenting nurse, but does not affect workload or cognitive load of medication nurses. Use of the InterFACE-AR system seems to improve teamwork performance but does not influence leadership performance of team leaders.

**Trial registration:**

ClinicalTrials.gov. Identifier: NCT06376643.

**Supplementary Information:**

The online version contains supplementary material available at 10.1186/s41077-026-00444-9.

## Background

The delivery of guideline-compliant clinical care to patients suffering from cardiac arrest requires effective teamwork, clear communication, and strong leadership [1–3]. Resuscitation teams comprised of trained healthcare providers often struggle to adhere to clinical guidelines for cardiac arrest [4]. Inconsistent adherence to guidelines may adversely affect survival and neurological outcomes. Balancing and distributing high provider workload and alleviating the cognitive load associated with making critical clinical decisions may help resuscitation teams improve clinical performance [5–7].

Cognitive aids are tools that present prompts aimed to encourage recall of critical information, and to increase the likelihood of desired behaviors, decisions, and outcomes [8–10]. Cognitive aids such as paper-based checklists, tablet or smartphone apps, voice guidance applications, and augmented reality-based guidance have all been used to support resuscitation teams during cardiac arrest [8, 10]. These tools have shown promising results when used by resuscitation teams, demonstrating improved adherence to guidelines and enhanced clinical performance during simulated neonatal, pediatric, and adult cardiac arrest [8, 10–13]. The impact of cognitive aids on provider workload, cognitive load and teamwork is less clear. While use of a digital app by team leaders may lower mental and physical demand during cardiac arrest [14], little is known about how cognitive aid use may affect other members of the resuscitation team [10]. Studies assessing the impact of cognitive aid use on teamwork and leadership during cardiac arrest or anesthetic emergencies have demonstrated mixed results, with some cognitive aids improving teamwork performance, and others negatively influencing team communication [10, 15]. A better understanding of these issues will inform future efforts to design and implement cognitive aids during cardiac arrest.

At many pediatric hospitals, providers use the American Heart Association (AHA) Pediatric Advanced Life Support (PALS) pocket card as a cognitive aid during resuscitation. While this pocket card provides evidence-based guidance, the text is small and difficult to read, not tailored to provider roles, and not designed to deliver critical information at the appropriate time. Our research team has designed and built a digital support system called ‘InterFACE-AR’ (Interconnected and Focused mobile Applications in the patient Care Environment with Augmented Reality-based guidance) to address these issues. InterFACE-AR is a multi-faceted clinical decision support system that incorporates augmented reality (AR)-based cognitive aids for the team leader and medication nurse during cardiac arrest [16]. The system is designed to deliver real-time, role-specific clinical guidance to the team leader and medication nurse via AR headset devices and enhance team situational awareness by projecting the relevant algorithm, prompts, and clinical data on a large Liquid Crystal Display (LCD) screen (i.e., TeamScreen) in the resuscitation room for the rest of the team. In this system, a tablet-based app (Guiding-Pad app) is used by the documenting nurse to document clinical data which is then used to trigger clinical prompts on the AR devices and TeamScreen. In this randomized controlled trial, we aimed to: (1) evaluate the effect of using the InterFACE-AR system vs. the AHA PALS pocket card on the workload and cognitive load of the team leader, medication nurse, and documenting nurse when managing a simulated cardiac arrest; (2) evaluate the effect of using the InterFACE-AR system vs. the AHA PALS pocket card on teamwork and leadership performance; and (3) describe the association between team leader workload and cognitive load with teamwork quality and leadership performance.

## Methods

### Study design

We conducted secondary analysis of data collected from a prospective, randomized controlled, multicenter trial comparing the use of the InterFACE-AR system to the AHA PALS pocket card during simulated pediatric cardiac arrest [17]. In the primary study, the objective was to evaluate the impact of the InterFACE-AR system on time to epinephrine administration and adherence to clinical guidelines. In this study, we evaluated the workload and cognitive load of resuscitation team members, leadership performance of the team leader, and teamwork performance of the resuscitation team. Institutional ethics review board approval was obtained at both study sites (i.e. Alberta Children’s Hospital and Geneva Children’s Hospital), and informed consent was acquired from all participants. This study was conducted and reported in compliance with the CONSORT reporting guidelines with simulation-based extensions [18, 19].

### Participants

Participants were voluntarily recruited from the emergency departments, intensive care units and inpatient units from two tertiary care pediatric hospitals in Calgary, Canada and Geneva, Switzerland. Participants were not reimbursed for participating in the study. Inclusion criteria for the team leader role included: being an attending physician, fellow, senior resident, or physician assistant in pediatric emergency medicine, intensive care, anesthesia or general pediatrics, or in adult emergency medicine. Inclusion criteria for the medication nurse and documenting nurse role included: being a nurse in pediatric emergency medicine, intensive care, anesthesia, surgery or general pediatrics. All participants were required to have obtained prior certification in basic life support training. Individuals were excluded if they had previously participated in the design or usability assessment of the InterFACE-AR system, if they were unable to perform the tasks assigned to them, or if they did not provide informed consent.

### Study intervention – the InterFACE-AR system

**Fig. 1:**
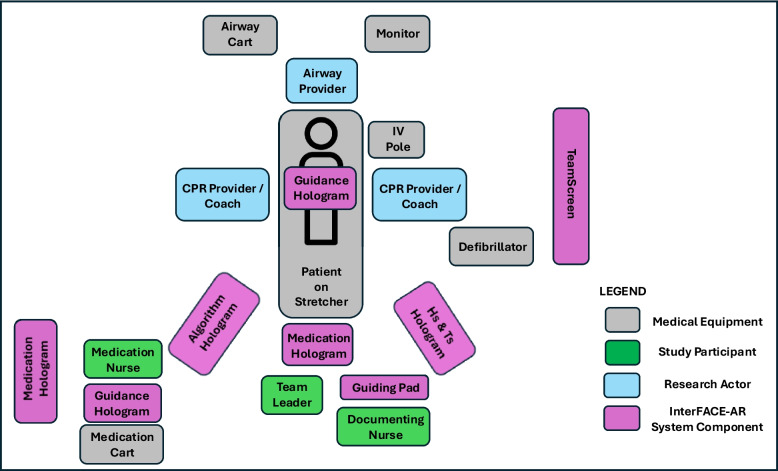
Room layout with holograms overlaid on clinical environment

The InterFACE-AR system comprises three key components: a tablet-based Guiding-Pad mobile app used by the documenting nurse, a TeamScreen display interface shown on a large LCD monitor, and two AR devices (Microsoft Hololens™ 2) for the resuscitation team leader and the medication nurse [17, 20] (supplemental file, eFigure 1a). (1) *Guiding-Pad app*: Based on designs from our prior studies, the Guiding-Pad app displays a split screen with the appropriate cardiac arrest algorithm on one side and the relevant stage of clinical care on the right side [20–22]. The documenting nurse actively enters patient data and completed tasks during the resuscitation, allowing the app to then send this data to the server which determines the clinical status and next steps (supplemental file, eFigure 1b). (2) *TeamScreen*: The TeamScreen offers a three-columned display showing the cardiac arrest algorithm on the left side, timers (for CPR and epinephrine), current tasks and next steps in the middle, and completed clinical tasks and medications on the right [20–23]. Placed on a wall in the room just to the left side of the patient, a 65-inch LCD screen provides a roadmap for clinical care, ensuring shared situational awareness amongst all team members (supplemental file, eFigure 1c). (3) *Augmented Reality Devices*: The two AR devices (Microsoft Hololens™ 2) provide real-time decision support and role-specific clinical guidance for the team leader and medication nurse. The AR devices display relevant timers (i.e., epinephrine and CPR timers for the team leader) and anticipatory guidance specific to the role of the provider wearing the AR device. For example, the team leader sees four holograms: the PALS algorithm matched to the ongoing rhythm/condition, current tasks and next steps with timers, a medication reference, and a list of reversible causes (supplemental file, eFigure 2). The medication nurse sees two holograms: one indicating which drugs (and doses) to prepare, and a second representing a medication reference card with relevant drugs and pre-calculated doses (supplemental file, eFigure 3). Figure 1 illustrates the room layout with holograms overlaid in the environment.

Amongst participants randomized to the control arm, team leaders were provided with a PALS Pocket Card to use as a resource. Medication nurses were also permitted to use their institutional medication references, and documenting nurses performed paper-based charting on local resuscitation forms. All nurses performing the documenting nurse role had prior experience with paper-based charting during cardiac arrest resuscitation.

### Study procedure and simulated scenario

Participants were recruited in groups of three to fulfill the roles of team leader, medication nurse, and documenting nurse for a resuscitation team. Three trained research actors played the scripted roles of CPR provider and CPR coach (2 actors, alternating between these two roles) and airway provider, to make a resuscitation team of 6 providers in total. The CPR Coach was responsible for operating the defibrillator. Research actors were trained to actively participate in the simulation scenario, respond to questions from team members, but not to offer clinical guidance or suggestions. Randomization of groups to the intervention arm or control arm occurred in a 1:1 ratio at the team level, stratified by study site, and with block size of two using an online random number generator (https://www.sealedenvelope.com). This strategy ensured equal distribution of intervention and control arms at each study site. Participants were only recruited to the study once.

After randomization, all groups received a video-based orientation to the study, simulation environment, and pediatric manikin (SimJunior™, Laerdal Corporation, Wappinger Falls, NY). Groups randomized to the intervention arm received an additional orientation to the InterFACE-AR system, including: (a) a short video describing the function of all its components; (b) a 5-min hands-on training with the relevant device (i.e. AR device for team leader and medication nurse, Guiding Pad app for the documenting nurse) guided by a checklist of tasks to complete; and (c) a brief verbal walk-through cardiac arrest scenario (4 min) allowing participants to use the assigned device and see the workflow during a scenario, but without performing actual tasks on the manikin. The entire InterFACE-AR system was setup and calibrated for participants by the research study team prior to the start of the study scenario. Participants randomized to the control arm did not participate in a verbal walk-through scenario; instead, they were provided the equivalent amount of time to review the AHA PALS pocket card and discuss their approach to managing cardiac arrest.

All teams performed a 12-min simulated pediatric cardiac arrest scenario, depicting a child with cardiac arrest secondary to hyperkalemia, progressing from pulseless electrical activity to pulseless ventricular tachycardia and finally to ventricular fibrillation (4 min per stage). Clinical progression was scripted and did not vary according to team interventions, thus ensuring a standardized scenario across all groups. The diagnosis of hyperkalemia was disclosed to the team 6.5 min into the scenario, providing all teams with the same amount of time to manage this issue. All resuscitation teams, regardless of study arm allocation, used a Zoll R-Series feedback defibrillator (Zoll Medical Corporation) with research actors performing the CPR coach and CPR provider roles. Direction to check pulse and rhythm, switch CPR providers and defibrillate was taken from the team leader. Scenarios were videotaped from a bird’s eye view from the foot of bed, with camera position and angle standardized across both study sites. Following scenario completion, all participants completed a survey collecting demographic data, workload, and cognitive load.

### Outcome measures

The primary outcome was provider workload, evaluated using the NASA-Task Load Index (NASA-TLX), a tool that assesses different dimensions of workload (i.e. mental, physical, temporal, performance, effort, and frustration), with established validity evidence from prior healthcare studies [6, 7, 24–27]. Total NASA-TLX or Raw TLX scores (RTLX) ranged from 0 to 100, with scores less than 40 representing low workload, scores from 40–60 representing moderate workload, and scores greater than 60 representing high workload. Secondary outcomes included provider cognitive load, leadership performance, and teamwork performance. Provider cognitive load was assessed using the Paas scale, comprised of one question with a 9-point Likert scale [28, 29]. NASA-TLX and Paas scores were collected from the team leader, medication nurse, and documenting nurse right after the simulation scenario. Leadership performance was assessed by video review using the CALM tool (Concise Assessment of Leader Management), a 17-item tool with 6 different categories of leadership, developed for use during simulated pediatric resuscitation, with a maximum total score of 61 [30]. Teamwork performance was assessed by video review using the TEAM (Team Emergency Assessment Measure) tool, a 12-item tool covering 3 key categories of teamwork behaviors, developed for assessing clinical resuscitation teams, with a maximum total score of 54 [31–33]. Four independent raters with clinical expertise in pediatric cardiac arrest and cardiopulmonary resuscitation were trained and calibrated to achieve an inter-rater reliability of greater than 0.75 at the end of training; two raters were assigned to each tool. All videos were reviewed in duplicate by the pair of raters assigned to each tool. Blinding was not possible due to the nature of the intervention.

### Sample size

The sample size was initially calculated to detect a clinically meaningful reduction in the time to first epinephrine dose as the primary outcome of the main study [17]. A total sample size of 18 (AHA PALS pocket card: 9 vs. InterFACE-AR: 9) teams was planned, which allowed detection of a large effect size (Cohen’s d = 1.32), with a two-sided significance level of 0.05 and a power of 0.8. Given the exploratory nature of this pilot study, the sample size was not intended to provide definitive conclusions but to estimate effect sizes and evaluate feasibility.

### Statistical analysis

All statistical analyses were conducted using R, version 4.5.0 (R Project for Statistical Computing). The demographic characteristics of team leaders, medication nurses, and documenters were summarized using descriptive statistics for each group. The mean difference between groups for all outcomes measures (NASA-TLX, Paas, TEAM and CALM scores) were calculated. Due to the small sample size, bootstrapping with 10,000 iterations was used to calculate the 95% confidence intervals (CIs), and permutation tests with 10,000 iterations were used to estimate *p*-values. Hedges’g was used as the effect size measure to account for small sample size. Associations between team leaders’ workload/cognitive load and teamwork or leadership performance were examined using Spearman’s rank correlation. Non-parametric methods were chosen due to the small sample size and the possibility that the assumptions underlying parametric tests were not met.

## Results

### Overview of data

A total of 18 simulation sessions were analyzed (AHA PALS pocket card: *n* = 9; InterFACE-AR: *n* = 9), involving 54 participants (18 team leaders, 18 medication nurses and 18 documenters). The demographic characteristics across all 3 roles were not significantly different between the AHA PALS pocket card and InterFACE-AR group. Attending physicians and fellows were pediatric emergency medicine specialists or trainees, apart from one fellow in general pediatrics recruited to the control arm. Four PALS instructors were recruited to the team leader role in the intervention arm, compared to one PALS instructor in the control group, but this was not a statistically significant difference (*p* = 0.24). (Supplemental File, eTable 1).

### NASA task load index and cognitive load

**Fig. 2:**
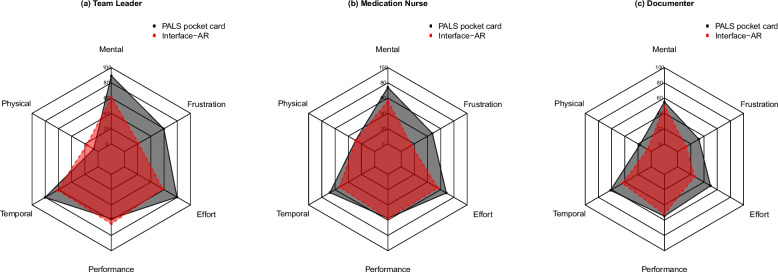
NASA-TLX subscale workload scores for the team leader, medication nurse, and documenting nurse

**Fig. 3:**
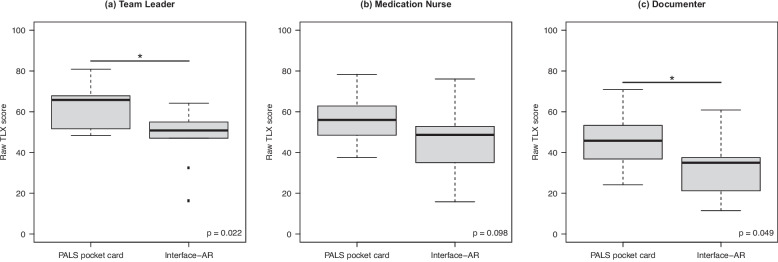
NASA-TLX raw workload (total) scores for the team leader, medication nurse, and documenting nurse, by study group

**Fig. 4:**
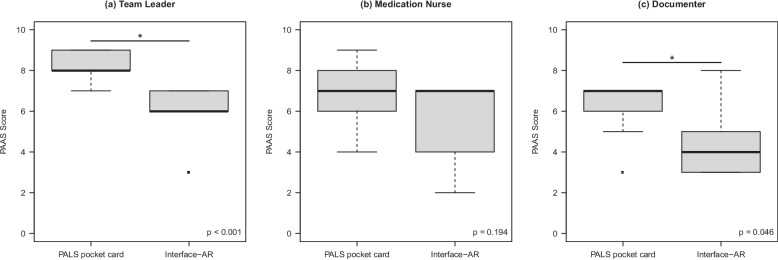
Cognitive load (Paas) scores for the team leader, medication nurse, and documenting nurse, by study group

Among team leaders, use of the InterFACE-AR system was associated with significantly lower scores for mental demand subscales (mean difference [MD]: −27.9, 95% CI: −45.8 to −12.0, *p* < 0.001), effort (MD: −21.8, 95% CI: −40.2 to −3.6, *p* = 0.044) and frustration (MD: −38.3, 95%CI: −60.6 to −14.6, *p* = 0.011) compared with the AHA PALS pocket card group. Conversely, physical demand was significantly higher (MD: 10.4, 95% CI: 2.8 to 20.1, *p* = 0.021). No significant differences were observed between the groups for temporal demand subscales (MD: −18.8, 95% CI: −35.8 to 2.0, *p* = 0.065) and performance (MD: 6.6, 95% CI: −14.5 to 27.2, *p* = 0.565). Team leaders using InterFACE-AR system also had significantly lower total raw TLX scores (MD: −15.0, 95%CI: −27.0 to −4.6, *p* = 0.022) and Paas scores (MD:—2.4, 95%CI: −3.6 to −1.4, *p* < 0.001) (Figs. 2a, 3a, and 4a; Supplemental Files, eTable 2).

For medication nurses, no significant differences were observed across NASA TLX subscales except for frustration, which was lower in InterFACE-AR group (MD: −31.6, 95% CI: −52.3 to −11.6, *p* = 0.013). Differences in total raw TLX (MD: −12.8, 95% CI: −26.4 to 1.0, *p* = 0.098) and Paas scores (MD: −1.3, 95% CI: −3.0 to 0.3, *p* = 0.194) did not yield statistical significance (Figs. 2b, 3b, 4b; Supplemental Files, eTable 3).

For documenters, the InterFACE-AR group reported significantly lower total raw TLX (MD: −13.7, 95% CI: −26.7 to −0.4, *p* = 0.049) and Paas (MD: −1.6, 95% CI: −2.8 to −0.1, *p* = 0.046) scores. Among NASA-TLX subscales, only effort was significantly lower (MD: −24.2, 95% CI: −41.9 to −5.8, *p* = 0.028); other subscales did not differ significantly between groups (Figs. 2c, 3c, 4c; Supplemental Files, eTable 4).

See Fig. 3 for Total Raw TLX scores by provider role and study group, and Fig. 4 for Pass scores by provider role and study group.

### Team Emergency Assessment Measure (TEAM) and Concise Assessment of Leader Management (CALM)

**Fig. 5:**
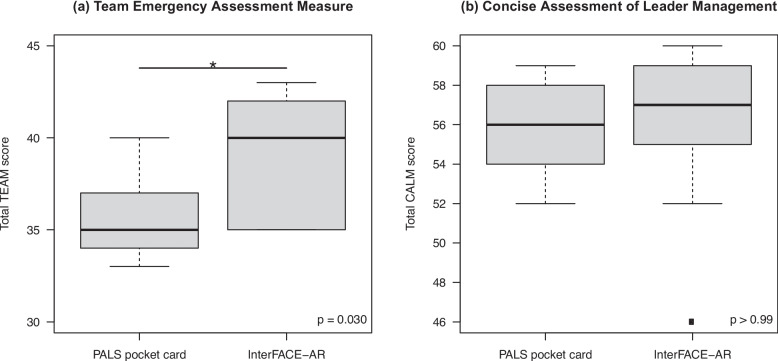
Teamwork (TEAM) and Leadership (CALM) performance of PALS Pocket Card vs. InterFACE-AR, by study group

Teams using InterFACE-AR system achieved significantly higher TEAM scores compared to those using PALS pocket card only (39.2 vs. 35.8, MD: 3.4, 95% CI: 0.8–5.9, *p* = 0.030). CALM scores did not differ significantly between groups (55.8 vs 55.7, MD: 0.1, 95% CI: −3.3 to 3.1, *p* = 0.99) (Fig. 5; Supplemental files, eTable 5). All videos were reviewed in duplicate by the pair of raters assigned to each tool. The intraclass correlation coefficient (ICC) was 0.90 for the CALM tool, and 0.81 for the TEAM tool.

### Association between teamwork and leadership performance and team leader workload and cognitive load

**Fig. 6:**
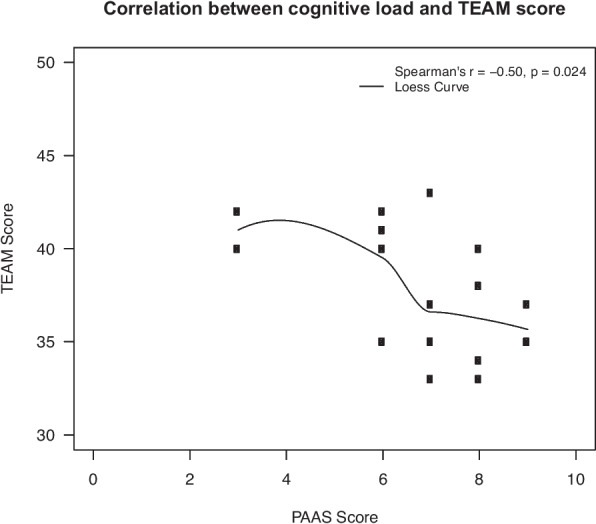
Correlation between team leader cognitive load and teamwork (TEAM) score

TEAM scores were negatively correlated with the team leader cognitive load measured by Paas scale (*r* =—0.5, *p* = 0.024) (Fig. 6). The correlation between TEAM scores and RTLX was not statistically significant (*r* =—0.39, *p* = 0.105). Leadership performance measured by CALM was not significantly associated with either Paas (*r* =—0.14, *p* = 0.562) or RTLX (*r* = −0.34, *p* = 0.160).

## Discussion

The dynamic nature of resuscitation presents a significant challenge for team leaders, who engage in complex cognitive process that result in extremely high workload and cognitive load [6, 7, 24], leading to poor decision-making and errors. Other resuscitation team members are often tasked with balancing multiple orders and prioritizing tasks, also challenging their ability to provide effective clinical care. Reducing the workload and cognitive load of team members by providing decision support during resuscitation can potentially improve quality of care during cardiac arrest. Our study suggests that the use of a multi-faceted digital decision support system, InterFACE-AR, may lower workload and cognitive load for the team leader and documenting nurse, as well as potentially improve teamwork performance compared to groups using the AHA PALS pocket card. Interestingly, we demonstrated a negative correlation between team leader cognitive load and overall teamwork performance; meaning lower team leader cognitive load is associated with higher teamwork performance scores. There was no reported association between team leader cognitive load and leadership performance.

Cognitive aids are decision support tools that present prompts to encourage recall of information, thus freeing up mental resources to increase the likelihood of desired behaviors [34]. Cognitive aids have been trialed in different forms for use during cardiopulmonary arrest, including pocket reference cards and digital apps. Simulation-based studies of cognitive aids used during cardiac arrest events have shown improved adherence to guidelines [22, 35–37], improved time to completing critical tasks [22, 35], and reduced rate of critical errors [36, 38]. Unfortunately, existing pocket reference cards have significant flaws—they all require providers to search through content to identify relevant information, which may potentially increase mental workload. Our study provides evidence to show that AR devices and an LCD display screen can directly address this issue by providing the appropriate clinical guidance without having to search through irrelevant content [11, 21]. AR can present individualized, role-specific guidance to reduce mental workload, cognitive load, and improve team performance; while an LCD display screen in the clinical environment can offer information to the entire team to improve teamwork. The current study evaluated the impact of the entire InterFACE-AR system as a whole on workload, cognitive load, and teamwork; future studies should aim to isolate the individual and combined effects of each component (i.e. AR only vs. LCD display only vs. InterFACE-AR system) to see which elements are contributing to improved outcomes.

Cognitive load describes “the amount of mental effort that is used from working memory while performing a cognitive task and/or when interacting with a system” [39]. In contrast, the NASA-TLX measures overall workload, which includes mental workload (i.e. how mentally demanding was the task?), but also physical demand (i.e. how physically strenuous was the activity?), temporal demand (i.e. how much time pressure did you feel?), effort (i.e. how hard did you have to work?), frustration (i.e. how irritated or stressed did you feel?) and performance (i.e. how successful were you?) elements [25, 27]. Two prior simulation-based studies reported moderate workload amongst team leaders participating in cardiac arrest scenarios with (RTLX = 52.7 [6]) and without a CPR Coach (RTLX = 54.1 [6]; RTLX = 56.1 [7]). By comparison, our study, in which both groups had a CPR Coach present, reported high team leader workload in the PALS pocket card group (RTLX = 62.2) and moderate workload (RTLX = 47.2) in the InterFACE-AR group.

Comparison of team leader workload across studies suggests that use of an AR-based cognitive aid reduces overall team leader workload, with the lowest workload value being that in team leaders using the InterFACE-AR group. One might (incorrectly) assume that using an AR device with holograms projected into the clinical space might increase team leader workload. In the design process, we specifically aimed to reduce the cognitive load and workload of team leaders by: (a) providing clinical prompts specific to their role; (b) simplifying the hologram display to reduce clutter; and (c) limiting interactivity with holograms. For example, the system did not require any data entry from the team leader to function effectively. We believe this approach to the design of the AR portion of the InterFACE-AR system contributed to the positive results we found for the team leader. Surprisingly, team leaders using the AR device reported a higher physical workload, despite the limited interactivity with holograms. We suspect this may be due to the need to physically scan the room to view all four holograms; future studies assessing visual gaze and fixations may shed light on this issue.

In contrast to the team leader’s AR display, the AR display for the medication nurse required some interactivity; specifically, the medication nurse was asked to press a ‘ + ’ button to indicate when a medication was drawn up and ready to be given and press tabs in the hologram to navigate between pages in the medication reference. The job of the medication nurse in the study is fairly task heavy, with many medications required to manage the patient. This may have contributed to the results we saw in our study, demonstrating no difference in the overall workload, the majority of workload subscales, and cognitive load of the medication nurses between groups. The documenting nurse is also a task-heavy job, requiring frequent charting or data entry (for the Guiding Pad app user) during the cardiac arrest event. While overall reduction in workload and cognitive load of participants using the guiding pad app is promising, closer examination of workload subscales showing no difference between arms (i.e. physical demand, temporal demand, performance, frustration) highlight opportunities for revisions of the app to improve overall impact. Future work involving the use of AR and tablet devices as cognitive aids should carefully consider the degree of interactivity required from the user as this may influence cognitive load and workload.

Effective teamwork and leadership are required to successfully manage patients suffering from cardiac arrest [1]. Introduction of new technology, such as tablet devices, AR devices and LCD screens, have the potential to disrupt team dynamics and interfere with communication. In pilot studies of earlier versions of the AR devices, some clinicians expressed concern that the display would be distracting and potentially have a negative effect on their ability to perform as a leader and communicate with team members. To our knowledge, our study is the first to assess team dynamics and leadership performance with use of AR-based cognitive aids for cardiac arrest. By demonstrating that teamwork improved with use of the InterFACE-AR system, we proved that AR technology could potentially be integrated into resuscitation teams and enhance how teams communicate, anticipate potential actions, and work together to complete tasks in a timely manner [31]. We showed a correlation between reduced team leader cognitive load and improved teamwork performance, but no correlation between team leader cognitive load and leadership performance. Perhaps this can be explained by the fact that leadership performance was not significantly different between groups. This is not entirely surprising given the sample of highly experienced providers, and that the InterFACE-AR system was not designed specifically to improve leadership performance. Given the pre-existing concerns expressed by many clinicians who tried the AR device during pilot testing, it is encouraging to see that use of the AR device did not negatively influence leadership behaviors of team leaders during cardiac arrest. This study provides preliminary proof of concept that AR devices could potentially be used during cardiac arrest without negatively affecting teamwork or leadership.

Our study has several limitations. We had a relatively small sample size and the study was not powered to detect significant differences for the primary outcome of this study (i.e., workload). Despite efforts in randomization, baseline imbalances could still exist, potentially introducing confounding biases. Additionally, there may be a clustered effect (i.e. the intervention may have different effects at the two sites). However, both are tertiary care pediatric centers in developed countries with similar care standards and protocols. Ideally, we would adjust for this clustered effect using a mixed-effect model, but due to the exploratory nature of this pilot study and the limited sample size, we lacked the power to implement such a model. Future studies with larger sample size are needed to confirm these findings and address these potential issues. Secondly, only half of the resuscitation team were participants, while the other half were research actors. Our research actors were trained to perform in a standardized fashion, and it is possible that this may have influenced team dynamics and leadership performance. In our study, the intervention group received a short verbal walk-through scenario as part of training, while the control group was given same amount of time to review cardiac arrest algorithms. This difference in preparation may have influenced the results. The intervention group also randomly had more team leaders with expertise as PALS instructors compared to the control group. While this was not deemed to be a statistically significant difference, it would be worthwhile further exploring the impact of PALS instructor status in a larger study. Finally, our study was conducted in a simulation-based context and not with real cardiac arrest patients. While our simulation teams are excellent at creating clinical environments that fully immerse participants, we recognize the fact that individual or team performance may differ slightly with real patients, thus potentially affecting the generalizability of our results. Future studies assessing the feasibility of implementing AR devices in the real clinical environment are necessary, particularly related to the time required for setup and device calibration.

## Conclusion

Use of a multi-faceted, AR-based decision support system during simulated pediatric cardiac arrest suggests a reduction in workload and cognitive load for the team leader and documenting nurse and improvement in teamwork performance compared to resuscitation teams using the AHA PALS pocket card. There were no significant differences in workload or cognitive load of medication nurses. Reduced team leader cognitive load was correlated with improved teamwork performance, but no association was demonstrated between team leader cognitive load and leadership performance. Future work is required to assess the feasibility and real-world impact of the InterFACE-AR system during pediatric cardiac arrest.

## Supplementary Information

Additional file 1: eFigure 1. Visual appearance of (a) Overall InterFACE-AR setup during simulated cardiac arrest scenario; (b) Guiding Pad app; and (c) TeamScreen. eFigure 2. First person point of view in Team Leader’s Augmented Reality Display (a) Main guidance hologram; (b) Algorithm (to the left of team leader); (c) Medication Card (at waist level, in front of team leader). eFigure 3. First person point of view in Medication Nurse’s Augmented Reality Display (a) Main guidance hologram; (b) Medication reference. eTable 1. Demographic Characteristics. eTable 2. NASA TLX and Paas Scores for the Team Leader. eTable 3. NASA TLX and Paas Scores for the Medication Nurse. eTable 4. NASA TLX and Paas Scores for the Documenting Nurse. eTable 5. TEAM and CALM Scores.

## Data Availability

Data available from authors upon request.

## Abbreviations

AR: Augmented reality
AHA: American Heart Association
CALM: Concise Assessment of Leader Management
CPR: Cardiopulmonary Resuscitation
InterFACE-AR: Interconnected and Focused mobile Applications in the patient Care Environment with Augmented Reality-based guidance
LCD: Liquid Crystal Display
RTLX: Raw Task Load Index
PALS: Pediatric Advanced Life Support
TEAM: Team Emergency Assessment Measure
