# Supplementary material for: Impact of an augmented reality-based decision support system on teamwork, leadership, provider workload and cognitive load during simulated cardiac arrest – a simulation-based randomized controlled trial
Source: Adv Simul (Lond). 2026 May 1;11:44. doi: 10.1186/s41077-026-00444-9 (PMC13281596; doi:10.1186/s41077-026-00444-9)
Supplement: Supplementary file 1 — Additional file 1: eFigure 1. Visual appearance of (a) Overall InterFACE-AR setup during simulated cardiac arrest scenario; (b) Guiding Pad app; and (c) TeamScreen. eFigure 2. First person point of view in Team Leader’s Augmented Reality Display (a) Main guidance hologram; (b) Algorithm (to the left of team leader); (c) Medication Card (at waist level, in front of team leader). eFigure 3. First person point of view in Medication Nurse’s Augmented Reality Display (a) Main guidance hologram; (b) Medication reference. eTable 1. Demographic Characteristics. eTable 2. NASA TLX and Paas Scores for the Team Leader. eTable 3. NASA TLX and Paas Scores for the Medication Nurse. eTable 4. NASA TLX and Paas Scores for the Documenting Nurse. eTable 5. TEAM and CALM Scores. [file 41077_2026_444_MOESM1_ESM.pdf]

Supplemental File

eFigure 1 - Visual appearance of (a) Overall InterFACE-AR setup during simulated cardiac arrest scenario; (b) Guiding Pad app; and (c) TeamScreen

(a)InterFACE-AR Setup during Cardiac Arrest Scenario (consent obtained by all individuals visualized in this photo)

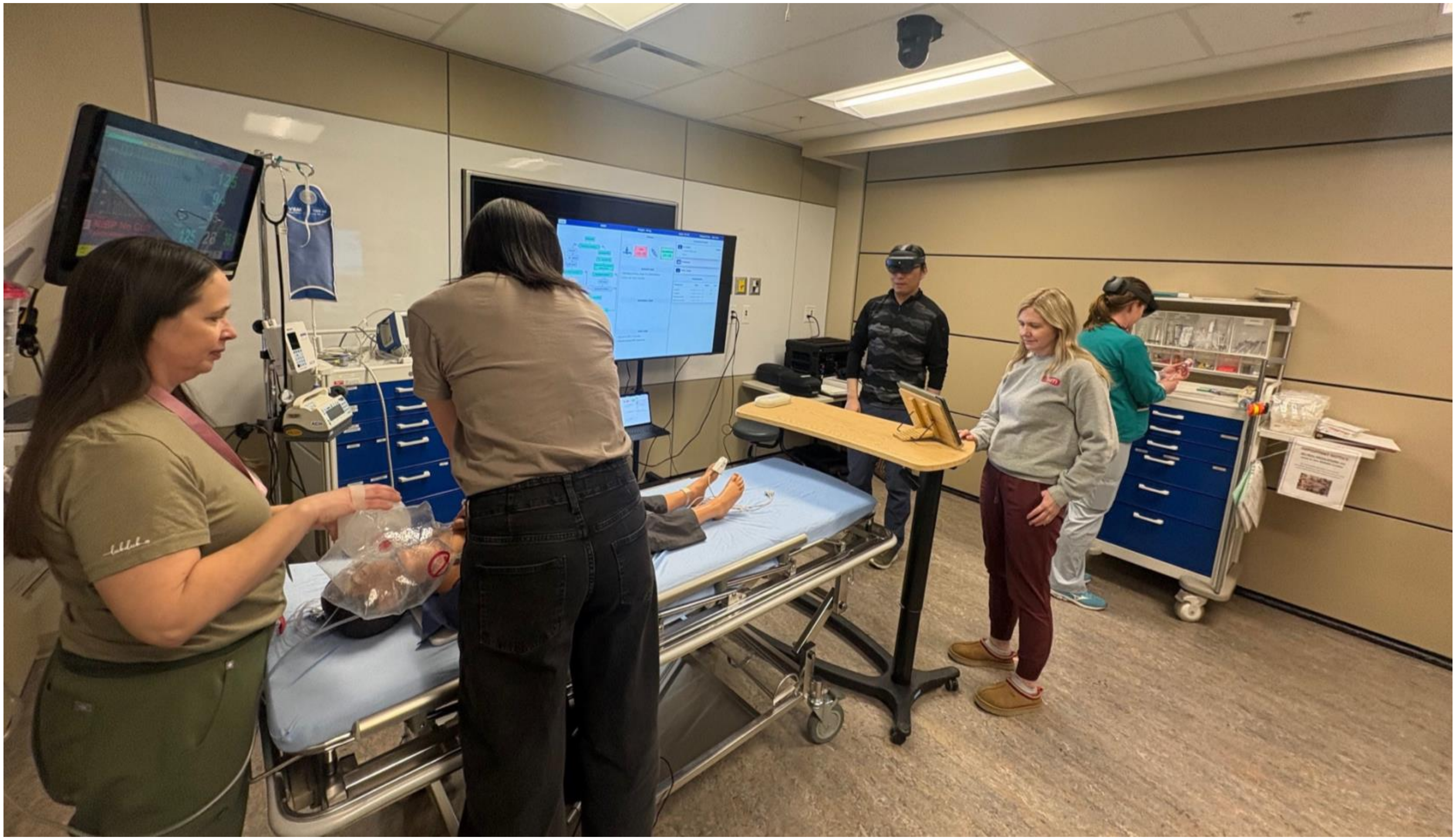

(b)Guiding Pad app

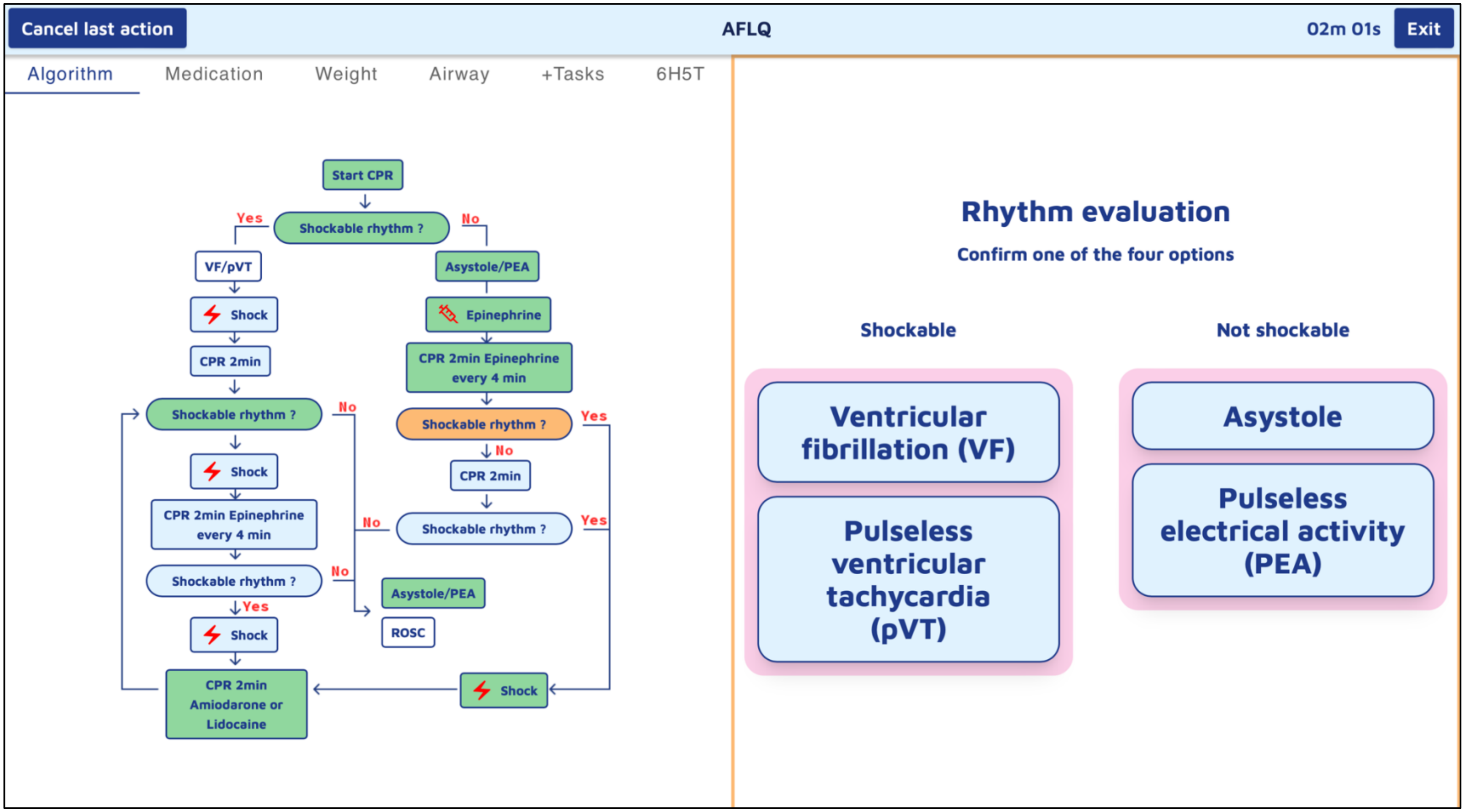

(c)TeamScreen

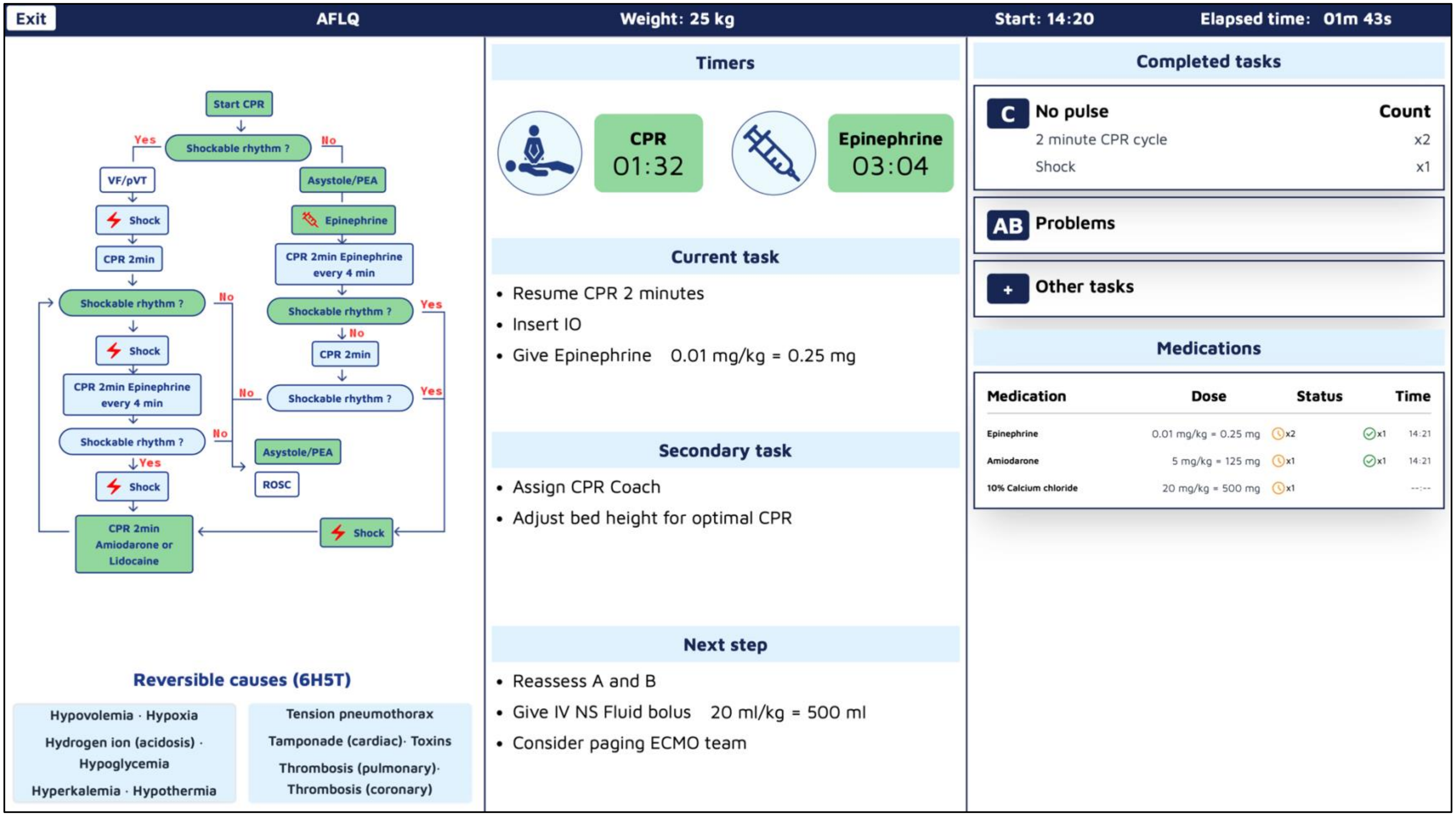

eFigure 2 – First person point of view in Team Leader's Augmented Reality Display (a) Main guidance hologram; (b) Algorithm (to the left of team leader); (c) Medication Card (at waist level, in front of team leader)

(a)Main guidance hologram

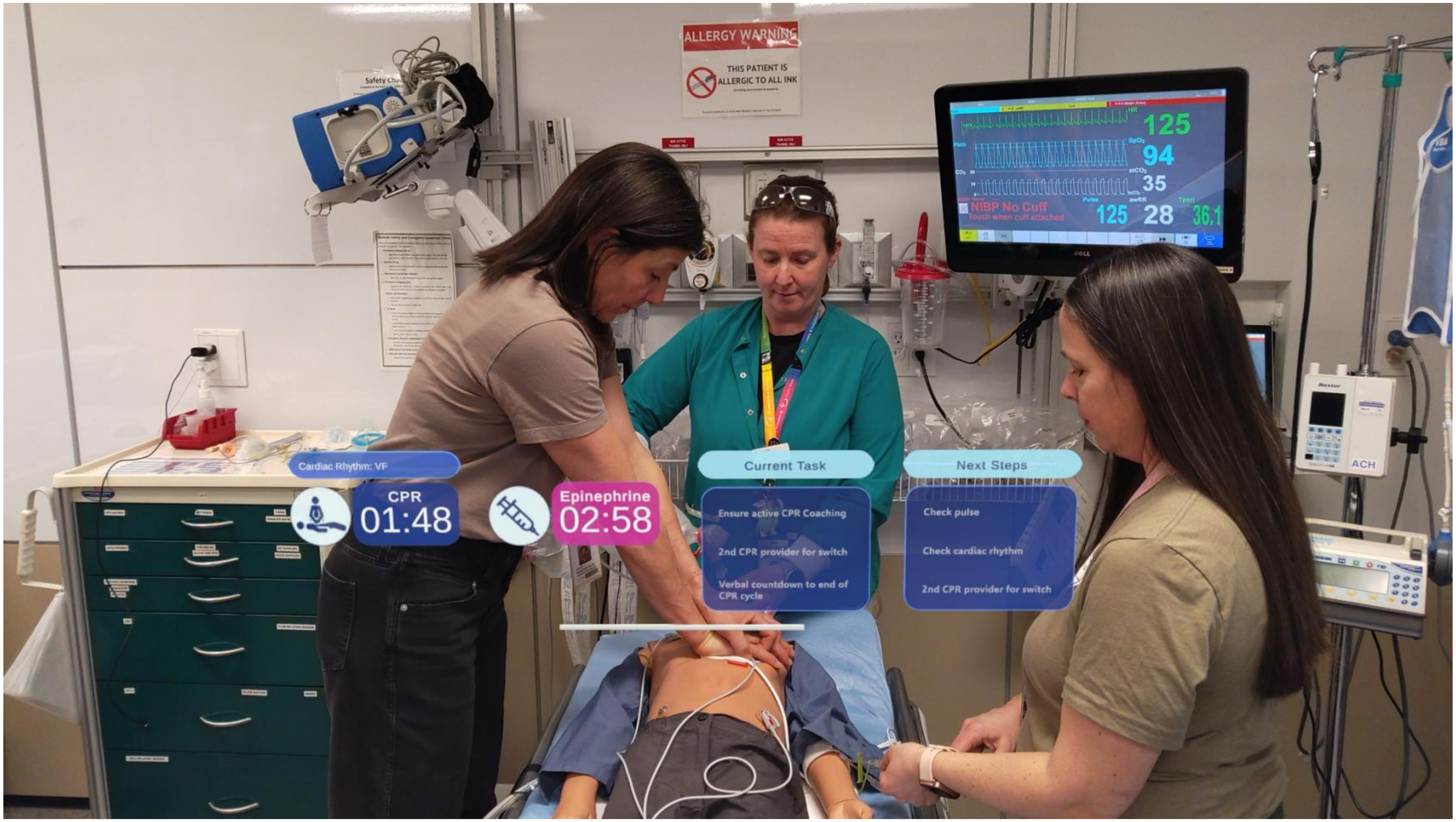

(b)Algorithm (to the left of team leader)

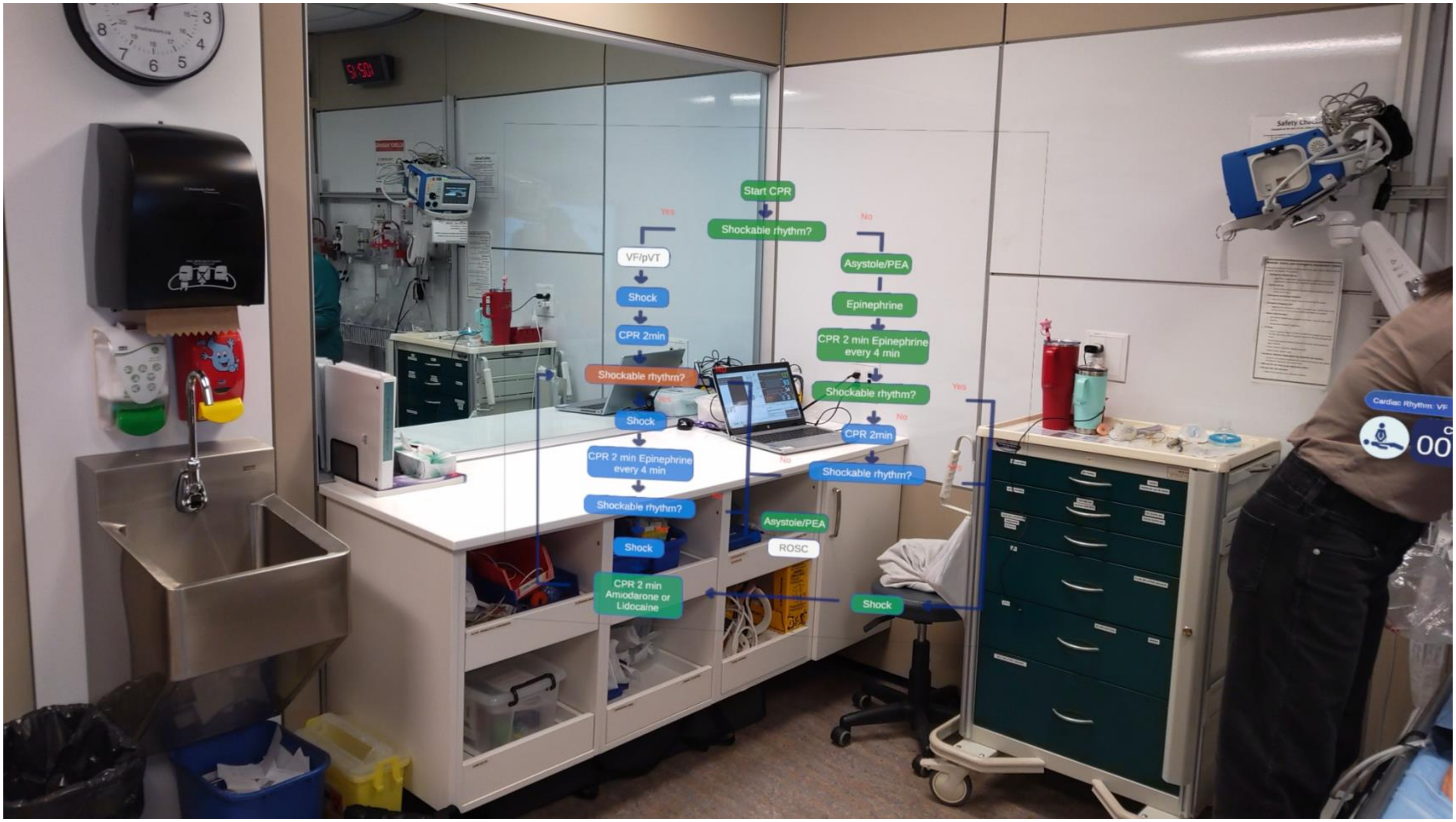

(c)Medication Card (at waist level, in front of team leader)

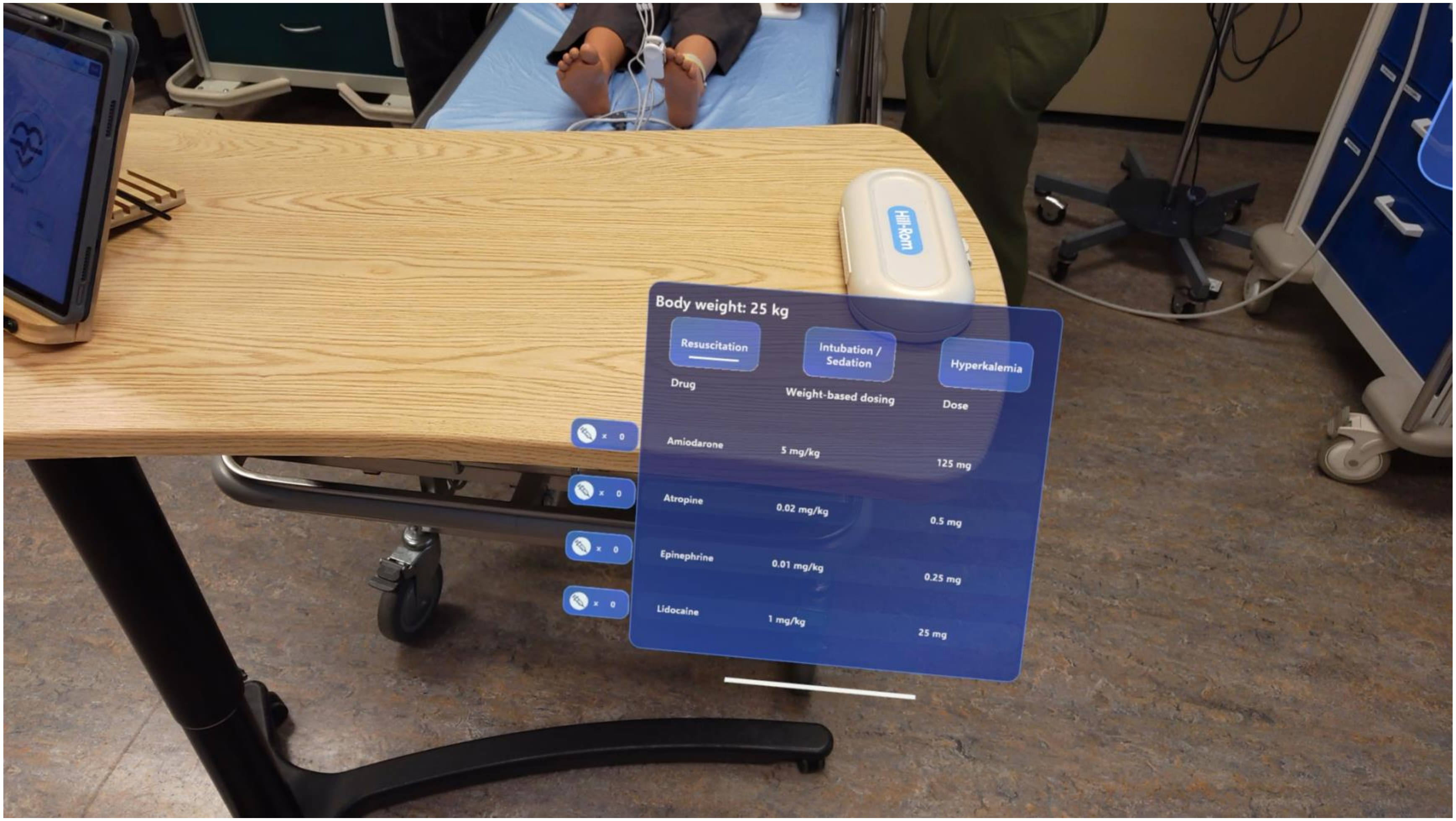

eFigure 3 – First person point of view in Medication Nurse's Augmented Reality Display (a) Main guidance hologram; (b) Medication reference

(a)Main guidance hologram

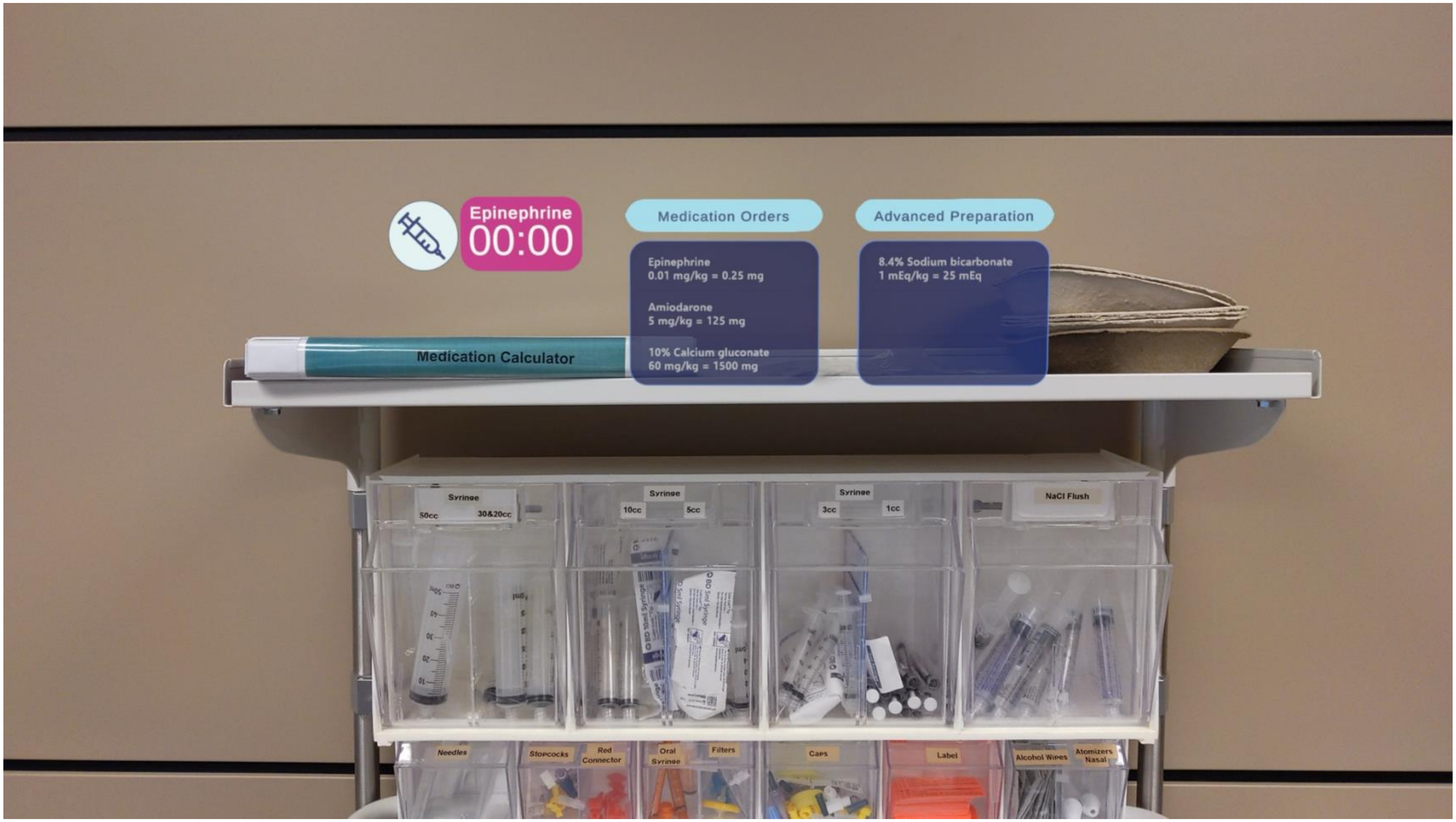

(b)Medication Reference

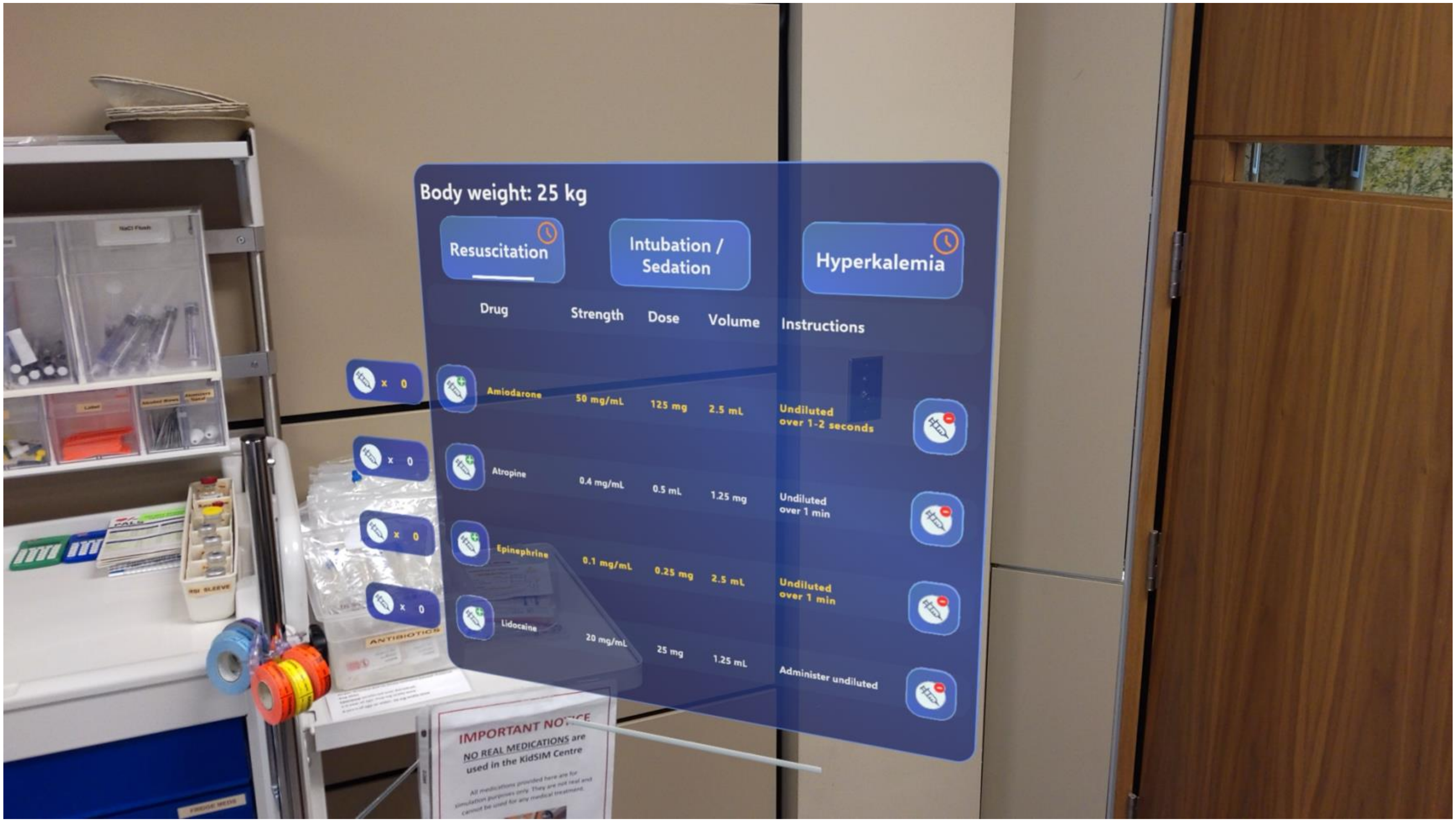

eTable 1: Demographic characteristics

Frequency (Percentage)	Team leader			Medication Nurse			Documenter		
	Control	InterFACE-AR	p-value	Control	InterFACE-AR	p-value	Control	InterFACE-AR	p-value
**Gender**									
Male	2 (22.2%)	3 (33.3%)	> 0.99	3 (33.3%)	0 (0%)	0.21	1 (11.1%)	0 (0%)	> 0.99
Female	7 (77.8%)	6 (66.7%)		6 (66.7%)	9 (100%)		8 (88.9%)	9 (100%)	
**Profession**									
Attending physician	3 (33.3%)	4 (44.4%)	0.31	0 (0%)	0 (0%)	0.58	0 (0%)	0 (0%)	> 0.99
Resident / Fellow	6 (66.6%)	3 (33.3%)		0 (0%)	0 (0%)		0 (0%)	0 (0%)	
Physician assistant	0 (0%)	2 (22.2%)		0 (0%)	0 (0%)		0 (0%)	0 (0%)	
Nurse	0 (0%)	0 (0%)		6 (66.7%)	8 (88.9%)		7 (77.8%)	7 (77.8%)	
Nurse Practitioner	0 (0%)	0 (0%)		3 (33.3%)	1 (11.1%)		2 (22.2%)	2 (22.2%)	
**Resuscitation training (BLS/ACLS/PALS/PEARS)**									
Instructor status	1 (11.1%)	4 (44.4%)	0.24	0 (0%)	1 (11.1%)	0.61	1 (11.1%)	1 (11.1%)	0.52
< 1 month	0 (0%)	1 (11.1%)		1 (11.1%)	1 (11.1%)		0 (0%)	2 (22.2%)	
1 - 6 months	1 (11.1%)	0 (0%)		1 (11.1%)	3 (33.3%)		5 (55.6%)	2 (22.2%)	
7 - 12 months	1 (11.1%)	0 (0%)		2 (22.2%)	0 (0%)		0 (0%)	0 (0%)	
> 12 months	6 (66.7%)	4 (44.4%)		5 (55.6%)	4 (44.4%)		3 (33.3%)	4 (44.4%)	
Number of resuscitation events involved (mean ± SD)	1.6 ± 1.6	2.0 ± 2.1	0.66	2.8 ± 4.8	0.8 ± 0.8	0.25	1.0 ± 1.0	0.9 ± 0.9	0.83
Number of simulated resuscitation events involved (mean ± SD)	1.9 ± 1.5	3.2 ± 3.2	0.29	3.1 ± 2.8	6.4 ± 8.1	0.27	3.0 ± 2.9	2.8 ± 2.5	0.88
Times of real CPR in the past year (mean ± SD)	0.4 ± 0.7	0.2 ± 0.4	0.47	0.7 ± 1.1	0.8 ± 0.8	0.83	0.6 ± 0.5	0.3 ± 0.5	0.22
Times of simulated CPR in the past year (mean ± SD)	1.0 ± 1.2	1.1 ± 1.3	0.87	1.8 ± 1.2	4.2 ± 5.1	0.2	1.9 ± 2.1	2.1 ± 1.9	0.83
**Experience with immersive technology**									
Never	7 (77.8%)	8 (88.9%)	> 0.99	8 (88.9%)	8 (88.9%)	> 0.99	8 (88.9%)	9 (100%)	> 0.99
Once a month or less	2 (22.2%)	1 (11.1%)		1 (11.1%)	1 (11.1%)		0 (0%)	0 (0%)	

Once a week to Once a month	0 (0%)	0 (0%)		0 (0%)	0 (0%)		1 (11.1%)	0 (0%)	
**Experience of using AR or VR for education**									
Never	7 (77.8%)	8 (88.9%)	> 0.99	7 (77.8%)	7 (77.8%)	> 0.99	6 (66.7%)	9 (100%)	0.21
Once a month or less	2 (22.2%)	1 (11.1%)		2 (22.2%)	1 (11.1%)		3 (33.3%)	0 (0%)	
Once a week to once a month	0 (0%)	0 (0%)		0 (0%)	1 (11.1%)		0 (0%)	0 (0%)	

eTable 2 – NASA TLX and Paas Scores for the Team Leader

Measure	PALS pocket card (Mean ± SD)	Interface AR (Mean ± SD)	Mean difference [95% CI]	Effect size (Hedges' d)	Permutation p-value
NASA_TLX					
Mental	89.1 ± 8.6	61.2 ± 27.0	−27.9 [−45.8, −12.0]	1.33	< 0.001
Physical	6.7 ± 6.0	17.1 ± 13.2	10.4 [2.8, 20.1]	-0.97	0.021
Temporal	80.3 ± 17.6	61.6 ± 21.9	−18.8 [−35.8, −2.0]	0.90	0.065
Performance	58.1 ± 24.8	64.7 ± 23.5	6.6 [−14.5, 27.2]	-0.26	0.565
Effort	79.4 ± 19.4	57.7 ± 22.3	−21.8 [−40.2, −3.6]	0.99	0.044
Frustration	59.6 ± 33.4	21.2 ± 18.6	−38.3 [−60.6, −14.6]	1.35	0.011
RTLX	62.2 ± 10.9	47.2 ± 14.6	−15.0 [−27.0, −4.6]	1.11	0.022
Cognitive load					
Paas Score	8.1 ± 0.8	5.7 ± 1.6	−2.4 [−3.6, −1.4]	1.81	< 0.001

RTLX: Raw Task Load Index (mean score for 6 subscales)

eTable 3 - NASA TLX and Paas Scores for the Medication Nurse

Measure	PALS pocket card (Mean ± SD)	Interface AR (Mean ± SD)	Mean difference [95% CI]	Effect size (Hedges' d)	Permutation p-value
NASA-TLX					
Mental	74.0 ± 11.2	56.2 ± 28.3	−17.8 [−37.1, 0.2]	0.79	0.105
Physical	28.9 ± 19.7	28.6 ± 25.8	−0.3 [−19.9, 20.0]	0.01	0.993
Temporal	67.7 ± 17.0	53.1 ± 33.3	−14.6 [−38.2, 7.6]	0.53	0.265
Performance	57.3 ± 27.6	57.8 ± 33.1	0.4 [−25.3, 27.1]	-0.02	0.983
Effort	68.7 ± 20.0	55.8 ± 31.5	−12.9 [−36.6, 9.3]	0.47	0.322
Frustration	47.4 ± 31.1	15.9 ± 12.0	−31.6 [−52.3, −11.6]	1.27	0.013
RTLX	57.3 ± 12.6	44.6 ± 18.0	−12.8 [−26.4, 1.0]	0.78	0.098
Cognitive load					
Paas Score	6.8 ± 1.8	5.4 ± 2.0	−1.3 [−3.0, 0.3]	0.70	0.194

eTable 4 – NASA TLX and Paas Scores for the Documenting Nurse

Measure	PALS pocket card (Mean ± SD)	Interface AR (Mean ± SD)	Mean difference [95% CI]	Effect size (Hedges' d)	Permutation p-value
NASA-TLX					
Mental	55.0 ± 22.8	49.2 ± 19.7	5.8 [−13.2, 23.8]	0.26	0.585
Physical	17.3 ± 24.4	5.3 ± 9.5	12.0 [-2.1, 29.8]	0.62	0.191
Temporal	61.7 ± 19.2	43.3 ± 24.6	18.3 [-1.7, 37.1]	0.79	0.098
Performance	54.7 ± 27.2	52.0 ± 37.1	2.7 [-25.5, 31.6]	0.08	0.871
Effort	49.9 ± 23.6	25.7 ± 17.1	24.2 [5.8, 41.9]	1.12	0.028
Frustration	32.2 ± 32.6	13.0 ± 16.2	19.2 [-2.4, 41.1]	0.71	0.135
RTLX	45.1 ± 15.3	31.4 ± 15.1	13.7 [0.4, 26.7]	0.86	0.049
Cognitive Load					
Paas Score	6.1 ± 1.4	4.6 ± 1.7	1.6 [0.1, 2.8]	0.92	0.046

eTable 5 – TEAM and CALM Scores

Measure	PALS pocket card (Mean ± SD)	Interface AR (Mean ± SD)	Mean difference [95% CI]	Effect size (Hedges' d)	Permutation p-value
TEAM	35.8 ± 2.4	39.2 ± 3.3	-3.4 [-5.9, -0.8]	1.12	0.030
CALM	55.7 ± 2.6	55.8 ± 4.5	-0.1 [-3.1, 3.3]	0.03	p > 0.99
